# Supplementary figures and images for: Supervised spike sorting feasibility of noisy single-electrode extracellular recordings: Systematic study of human C-nociceptors recorded via microneurography
Source: PLoS One. 2025 Sep 26;20(9):e0329537. doi: 10.1371/journal.pone.0329537 (PMC12469167; doi:10.1371/journal.pone.0329537)

A1

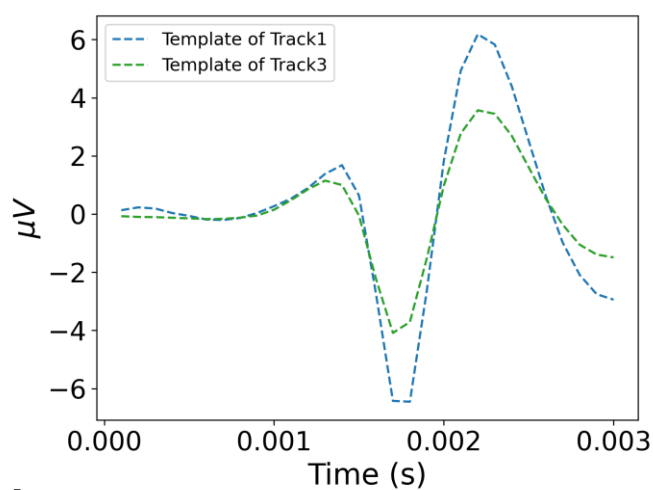

A2

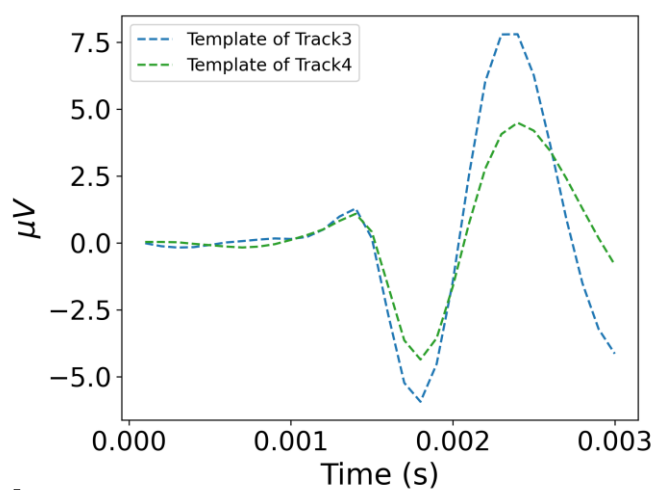

A3

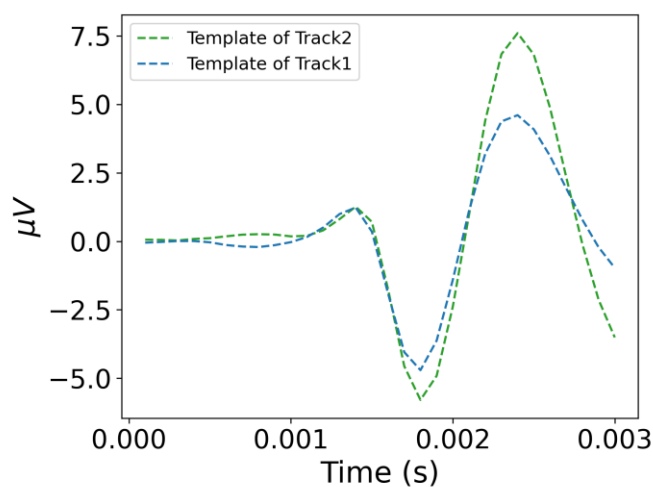

A4

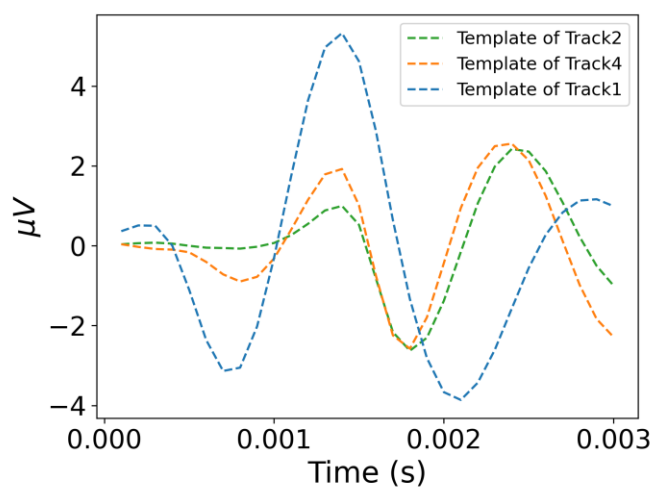

A5

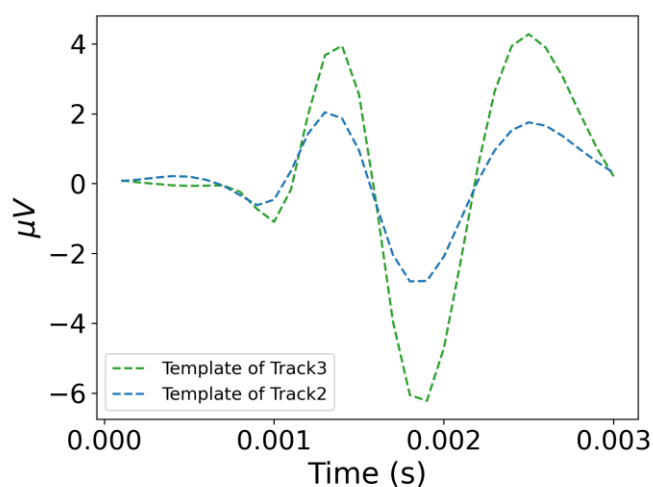

A6

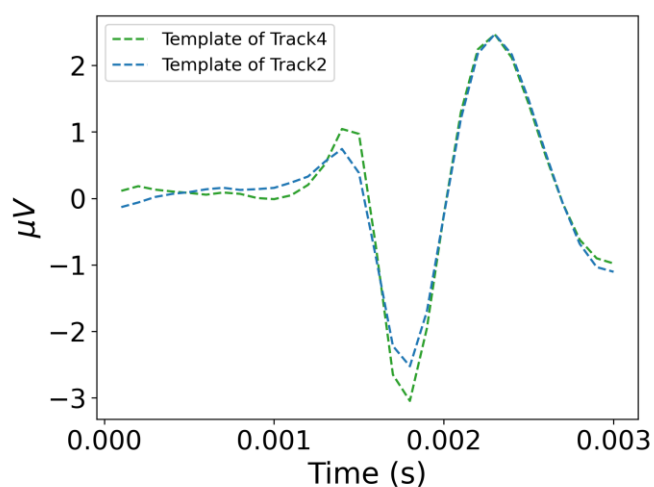

A7

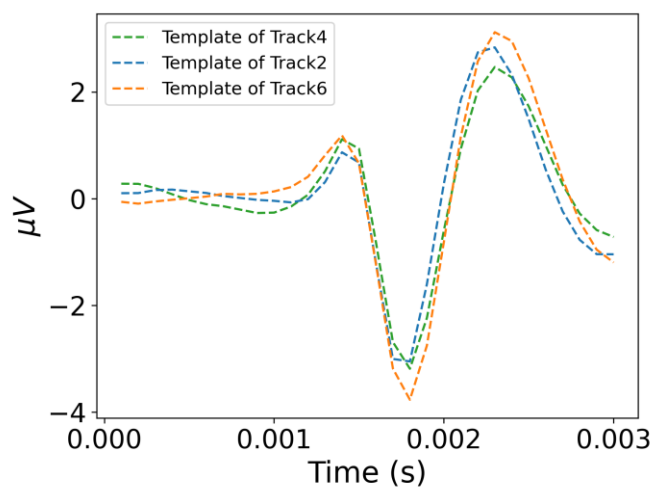

A8

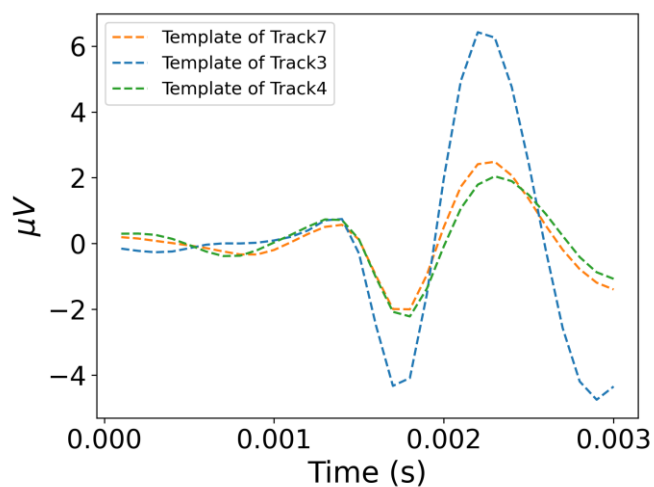

# A9

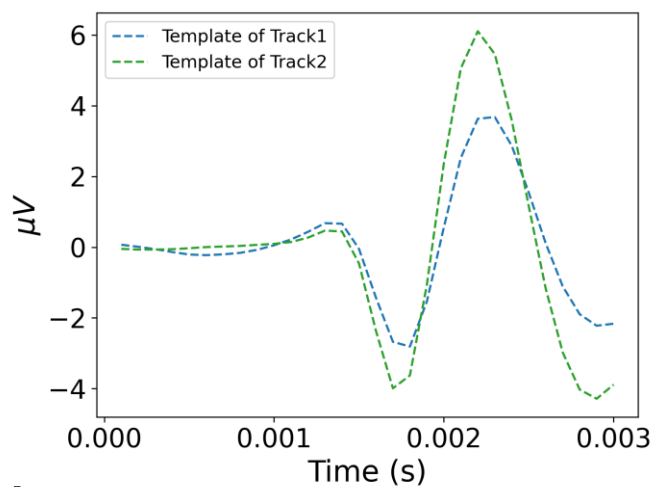

# A10

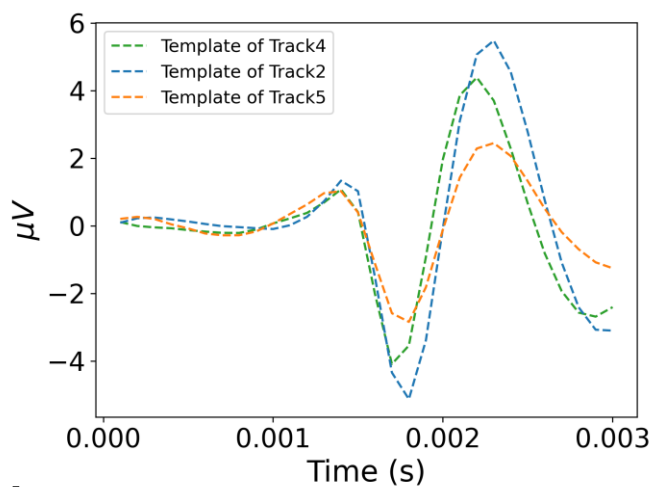

# A11

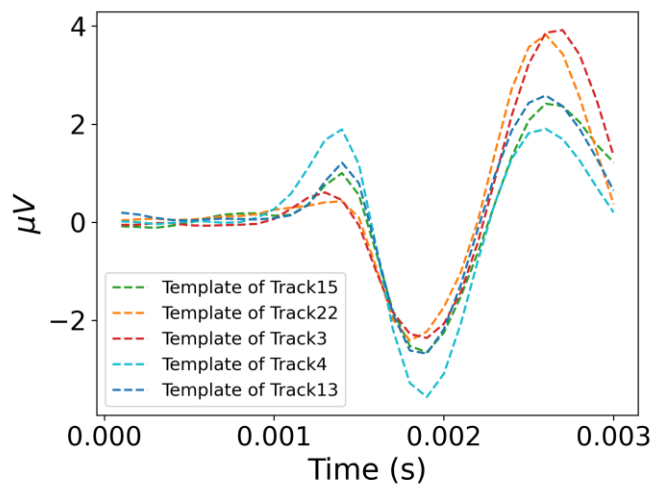

# A12

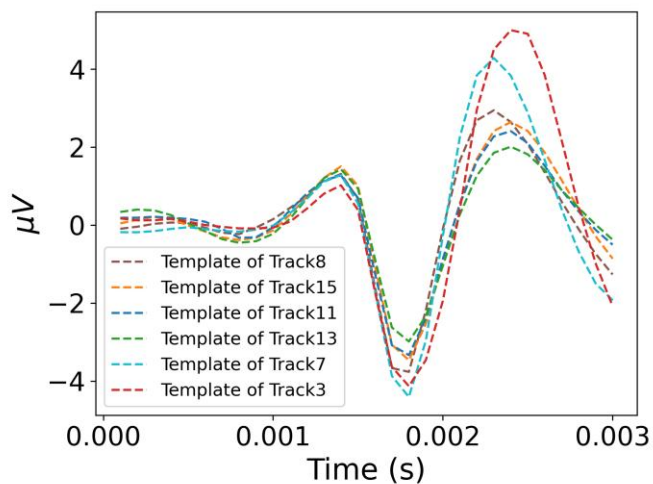

# A13

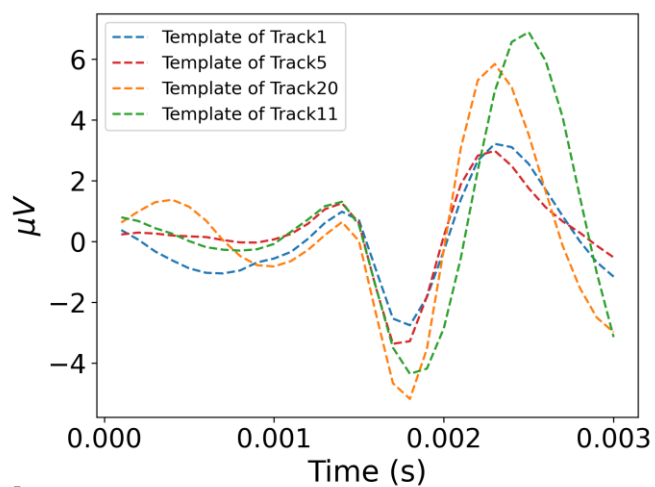

# A14

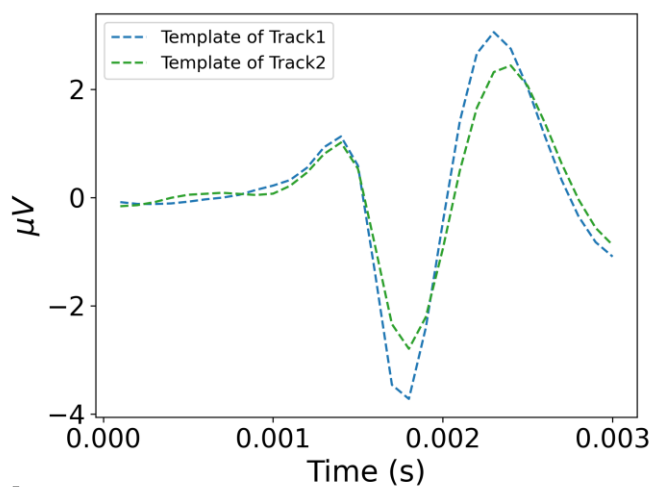

# A15

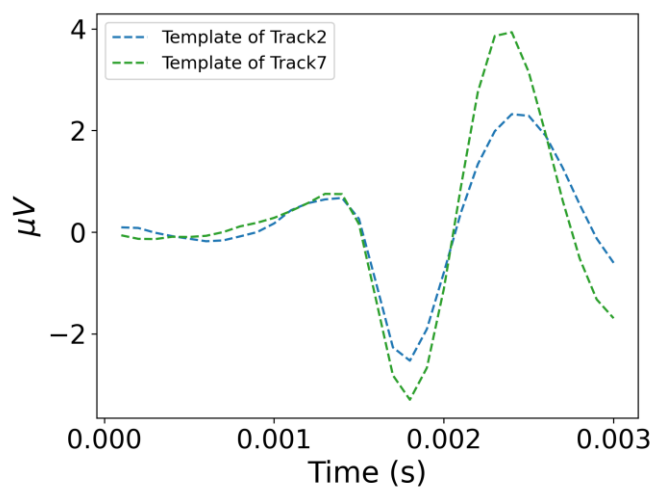

# A16

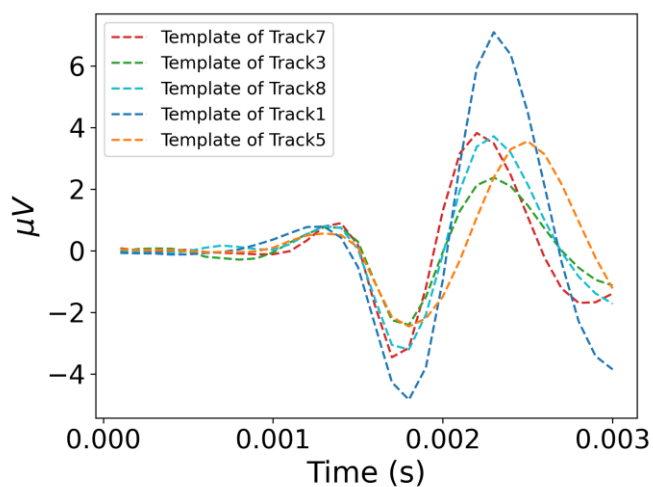

**A17**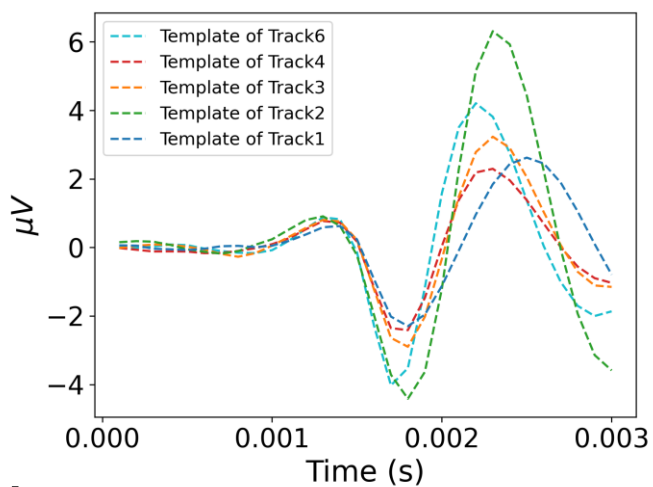**A18**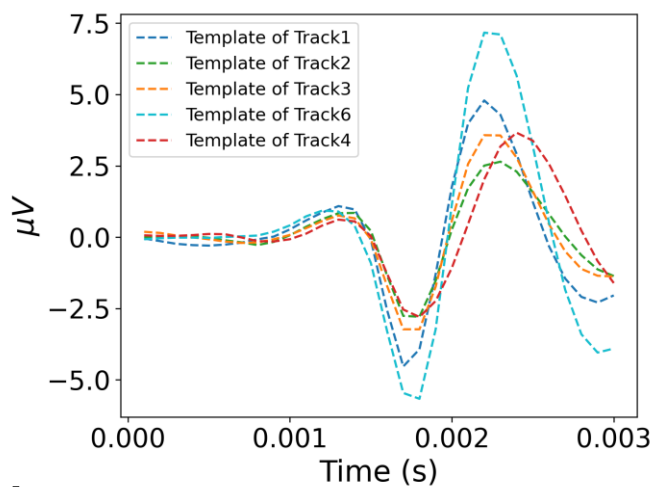**A19**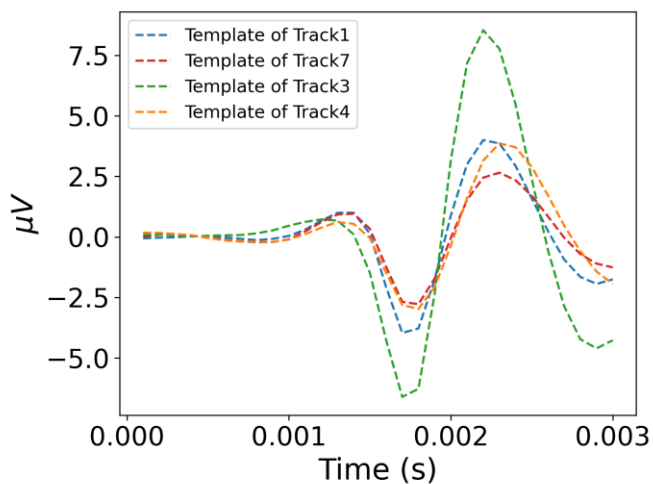**A20**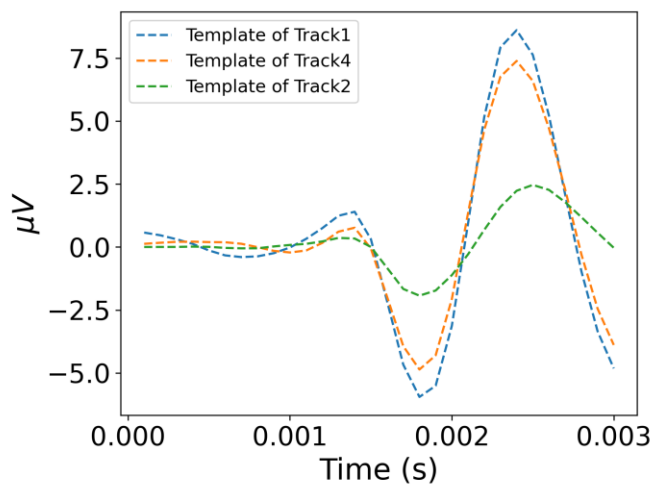**A21**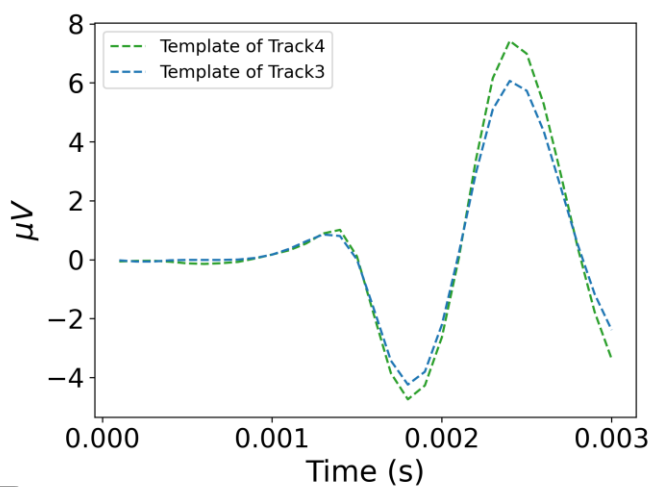**A22**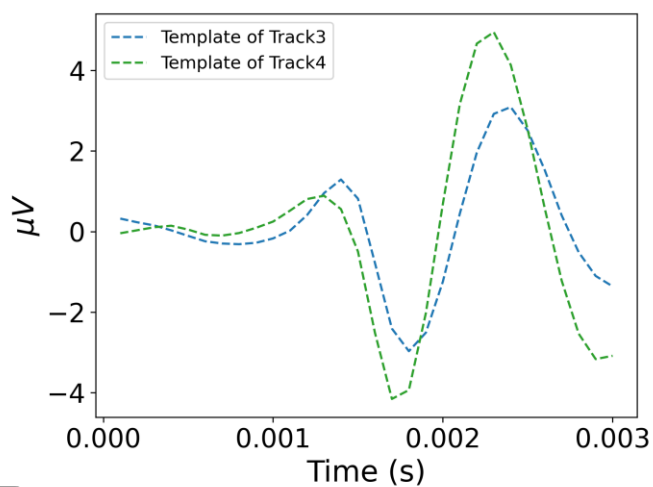**B1**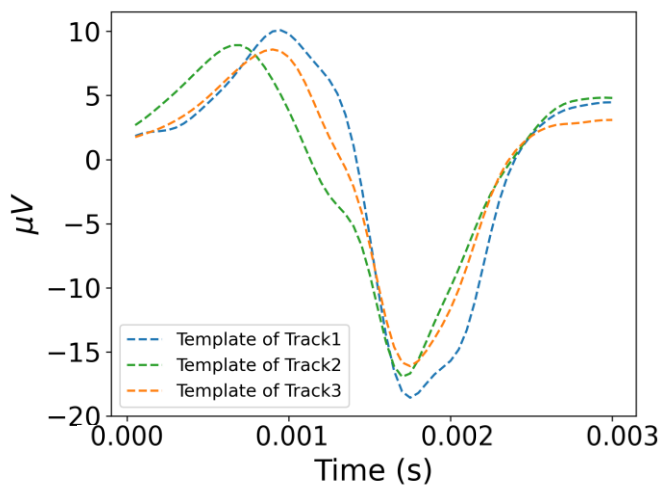**B2**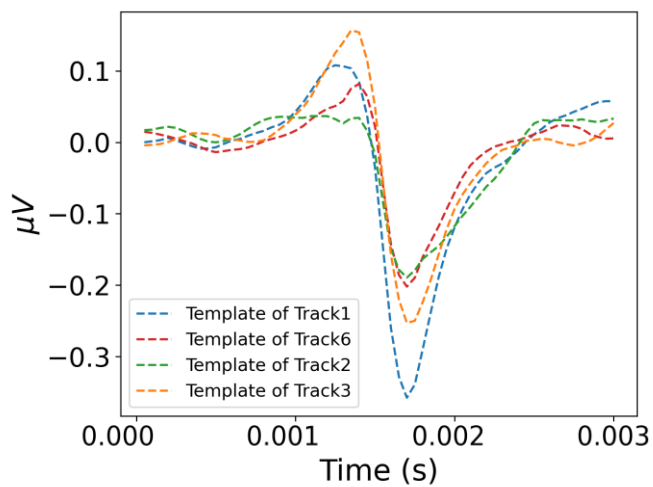

B3

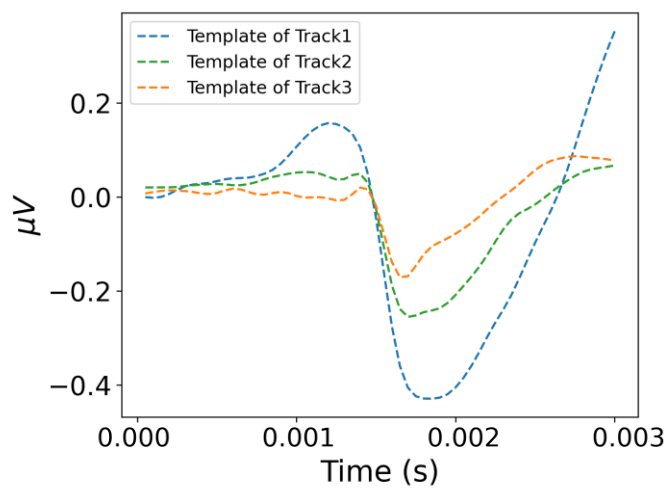

B4

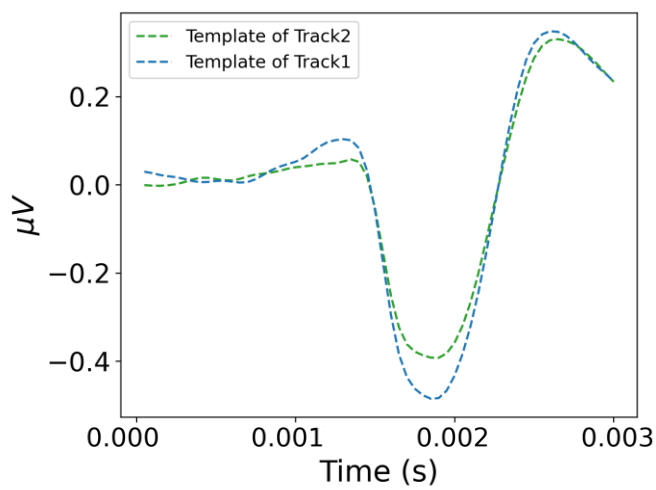

Supplement: S5 File — The spike templates were computed by averaging all tracked spikes after aligning them to the time point of their maximum negative peak. These templates represent characteristic waveforms for each identified track across recordings and give insights into morphological differences. (PDF) [file pone.0329537.s005.pdf]
