# Supplementary material for: Supervised spike sorting feasibility of noisy single-electrode extracellular recordings: Systematic study of human C-nociceptors recorded via microneurography
Source: PLoS One. 2025 Sep 26;20(9):e0329537. doi: 10.1371/journal.pone.0329537 (PMC12469167; doi:10.1371/journal.pone.0329537)

S6.1 Figure. Cumulative explained variance ratio for dataset A1. This figure illustrates the cumulative explained variance ratio for the first eight principal components computed for dataset A1. The visualization shows how much of the total variance in the spike waveform is captured as additional components are included, providing insight into the dimensionality required to represent the spike effectively.

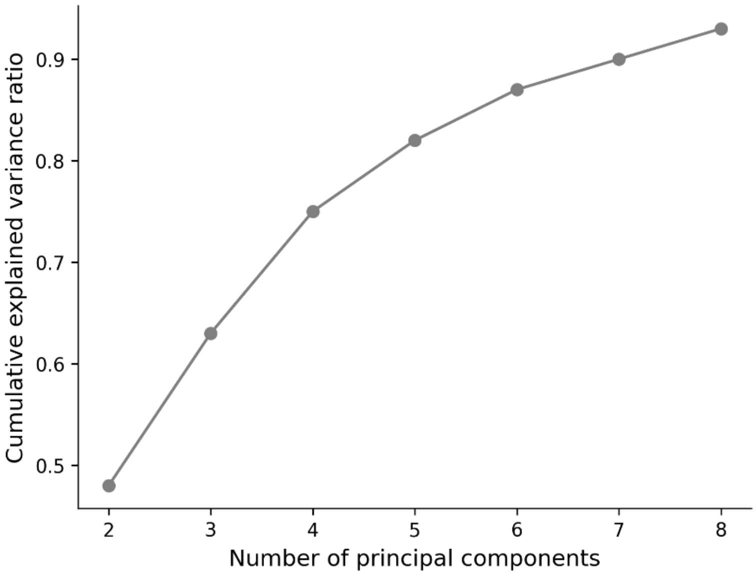

S6.2 Figure. Cumulative explained variance ratio for dataset A3. This figure illustrates the cumulative explained variance ratio for the first eight principal components computed for dataset A3. The visualization shows how much of the total variance in the spike waveform is captured as additional components are included.

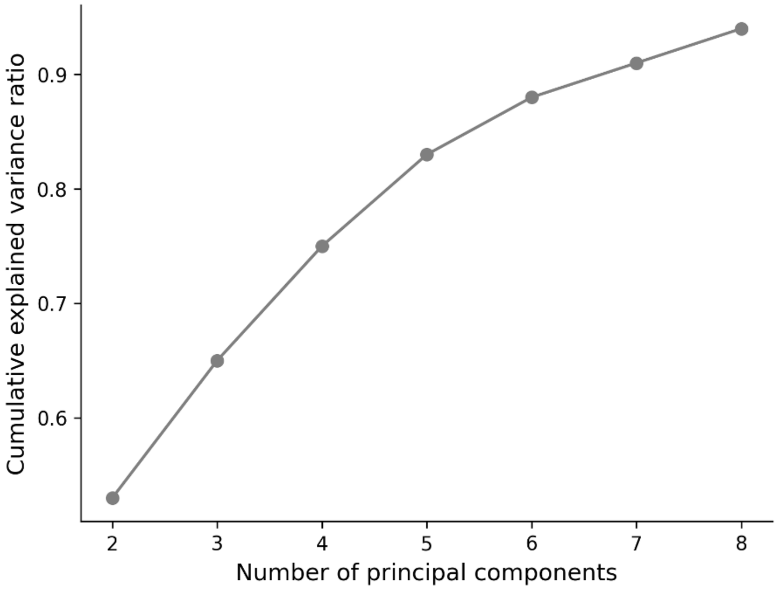

Supplement: S6 File — The cumulative explained variance ratio for principal component counts ranging from 2 to 8, visualized separately for datasets A1 (S6.1) and A3 (S6.2). The plots illustrate how the proportion of total variance captured increases with the number of PCA components, providing insight into the dimensionality required to represent the spike waveform effectively. (PDF) [file pone.0329537.s006.pdf]
