# Supplementary material for: Supervised spike sorting feasibility of noisy single-electrode extracellular recordings: Systematic study of human C-nociceptors recorded via microneurography
Source: PLoS One. 2025 Sep 26;20(9):e0329537. doi: 10.1371/journal.pone.0329537 (PMC12469167; doi:10.1371/journal.pone.0329537)
Supplement: S15 File — To illustrate the tracking process, this file includes two screenshots, one from Dapsys and one from SpikeSpy. Both software tools implement similar tracking mechanisms to extract vertically aligned spike waveforms during microneurography recordings, providing experimental ground truth for subsequent analysis. (PDF) [file pone.0329537.s015.pdf]

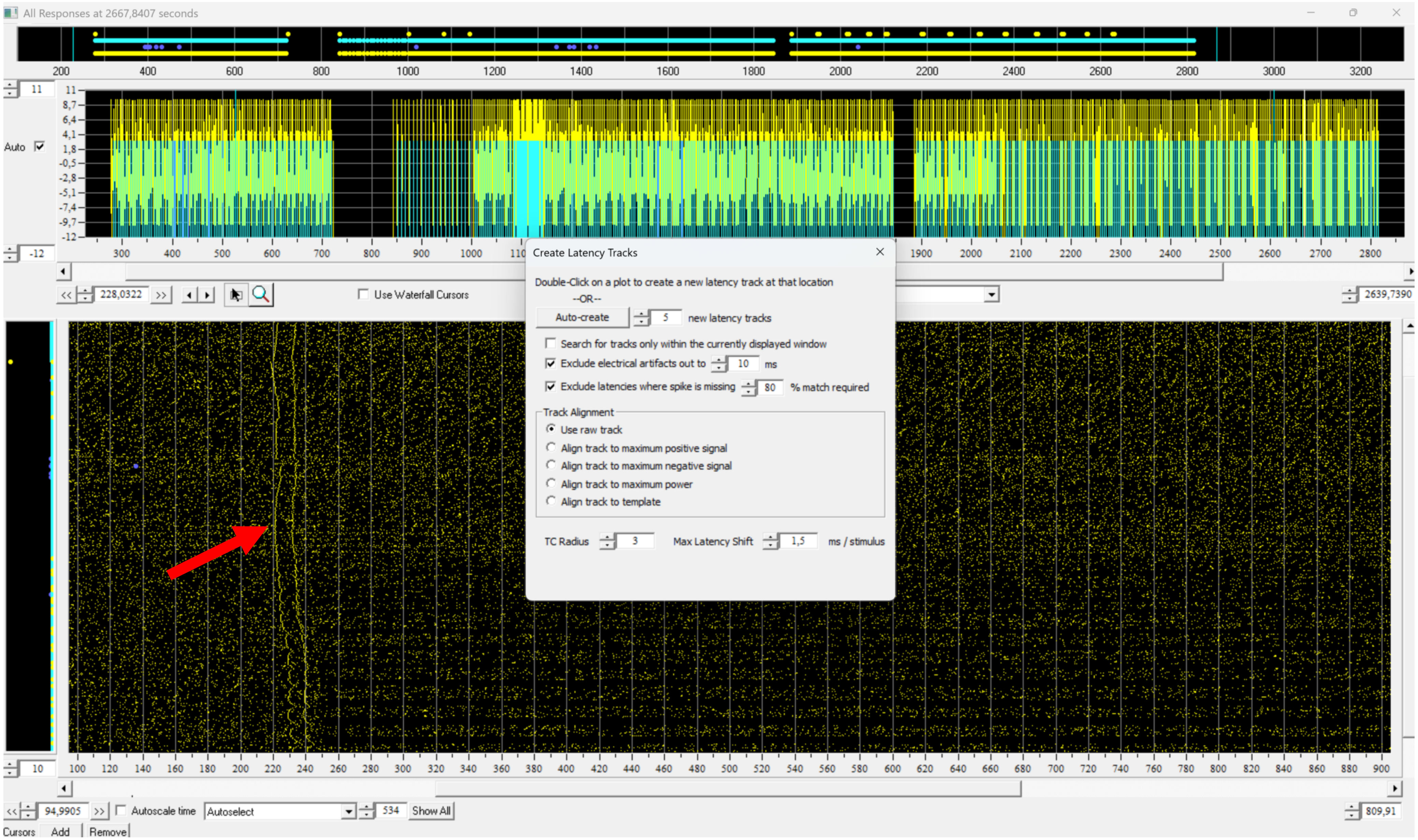


**S15.1 Figure. Screenshot of Dapsys for offline tracking.** The image shows a recording in Dapsys. The internal tracking algorithm can identify and mark the two distinct tracks, indicated by the red arrow. These tracks serve as experimental ground truth for subsequent spike classification and analysis.

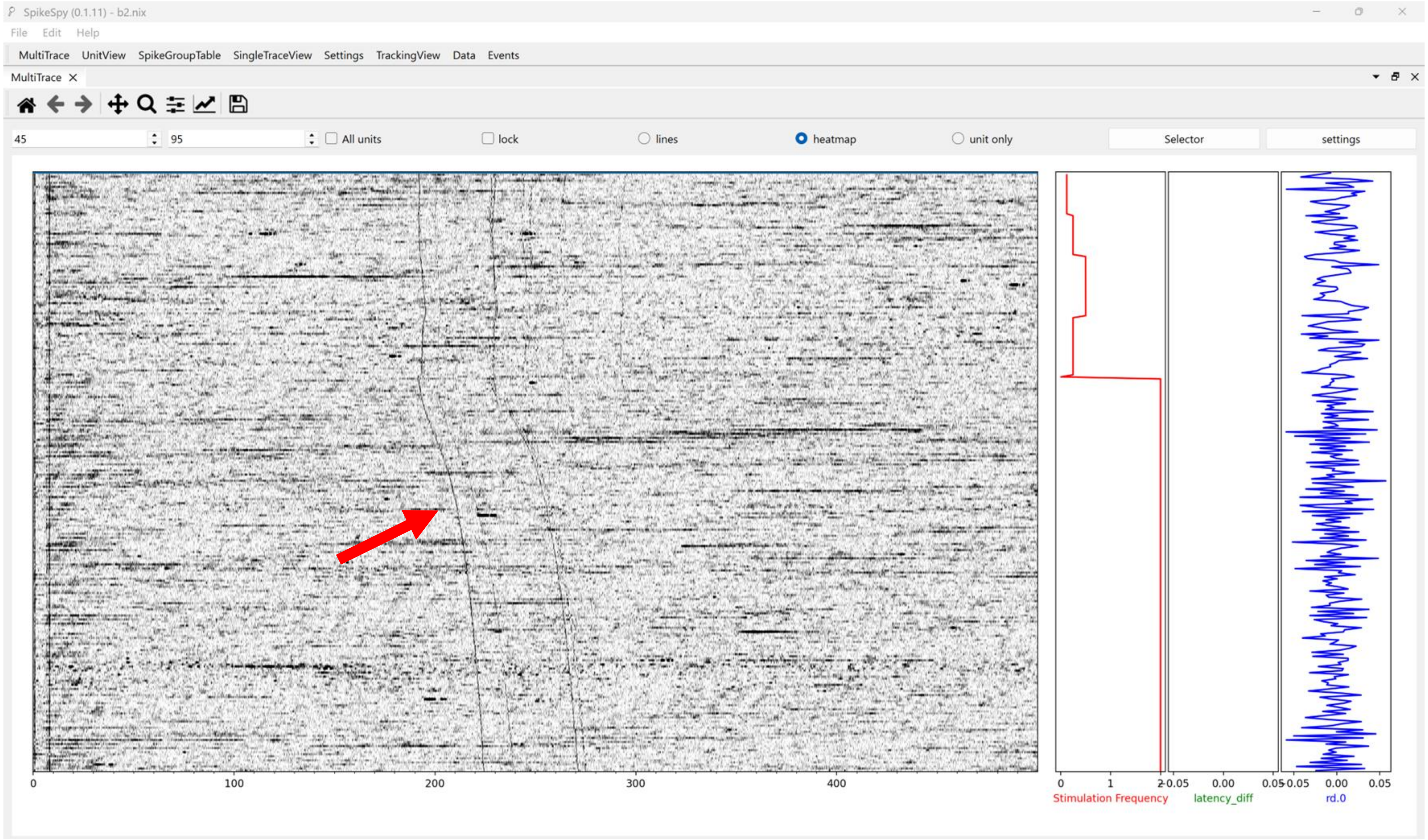


**S15.2 Figure. Screenshot of SpikeSpy for offline tracking.** The internal tracking algorithm can identify and mark the distinct tracks, indicated by the red arrow. These tracks serve as experimental ground truth for subsequent spike classification and analysis.
